# Prognostic Value of Systemic Immune‐Inflammation Index (SII) in Hospital Readmission Following Acute Myocardial Infarction: A Four‐Year Analysis

**DOI:** 10.1155/crp/6967879

**Published:** 2026-05-20

**Authors:** Yasaman Borghei, Bahare Gholami-Chaboki, Nasibe Goli, Fatemeh Baharvand, Arsalan Salari

**Affiliations:** ^1^ Department of Cardiology, Cardiovascular Diseases Research Center, Heshmat Hospital, School of Medicine, Guilan University of Medical Sciences, Rasht, Iran, gums.ac.ir

**Keywords:** coronary artery disease, inflammation, myocardial infraction, outcome

## Abstract

**Background:**

Acute myocardial infarction (AMI) initiates an inflammatory response essential for cardiac repair. Several novel inflammatory markers have been identified as indicators of systemic inflammation, including the systemic immune‐inflammation index (SII). This study aimed to evaluate the relationship between SII levels and readmission rates in AMI patients.

**Methods:**

This retrospective cohort study analyzed 1147 AMI patients admitted between 2019 and 2021. The optimal SII cutoff was determined as 694.3 × 10^9^/L, categorizing patients into high SII (≥ 694.3) and low SII (< 694.3) groups. The association between SII levels and readmission risk was assessed using a logistic regression model.

**Results:**

Over 4 years, 454 patients were readmitted, with higher readmission rates (34.4%) observed in the high SII group. Statistically significant differences were noted in primary outcomes, with higher SII patients experiencing increased incidence of major adverse cardiovascular events (MACEs) (*p* < 0.05). The Cox proportional hazards model indicated that individuals with prior CABG had a 40% higher risk of readmission.

**Conclusion:**

Although patients with high SII demonstrated higher hospital readmission rates, primarily due to recurrent MI, no statistically significant association was observed.

## 1. Introduction

Cardiovascular diseases (CVDs) remain the leading cause of mortality worldwide, accounting for a significant proportion of global deaths. In Iran, approximately 43% of all mortalities are attributed to CVDs. Over the past few years, both the incidence and prevalence of these conditions have been steadily rising, highlighting an urgent public health concern [1]. The primary contributor to cardiovascular death is acute myocardial infarction (AMI) [2]. AMI initiates an inflammatory response essential for cardiac repair. However, excessive inflammation can contribute to adverse left ventricular remodeling and the progression of heart failure (HF). Beyond localized myocardial inflammation, patients with AMI also exhibit an amplified systemic inflammatory response [3].

It has been shown that inflammation plays a fundamental role in both the development and persistence of atherosclerosis [4]. Routine blood tests including complete blood cell count (CBC) are used to evaluate inflammatory processes and are often useful in the early diagnosis of several diseases [5, 6]. Several new inflammatory parameters have been identified that are indicative of the systemic inflammatory response [7]. These new indicators include the main components of previously known inflammatory markers, such as neutrophils, monocytes, lymphocytes, and platelets [8, 9]. These indicators are presented as prognostic factors of possible mortality in various CVDs and non‐CVDs. One of these indexes is the systemic immune‐inflammation index (SII) [10, 11]. It has been proposed that this index incorporates three key inflammatory cell types: platelets, neutrophils, and lymphocytes, and can be determined using the following equation: platelet count × neutrophil count/lymphocyte count [12].

Hospital readmissions among AMI patients are common, costly, and often preventable [13]. Studies indicate that approximately one in six AMI patients is rehospitalized within 30 days [14]. Identifying individuals at high risk of readmission allows healthcare providers to implement targeted interventions, ensuring that patients who would benefit most receive intensive preventive measures. This approach also helps optimize the allocation of limited healthcare resources, enhancing the effectiveness of intervention strategies [15]. Although SII has been extensively studied in CVDs and shown to predict mortality, adverse outcomes, and long‐term prognosis, its potential role in predicting hospital readmission after AMI has not been adequately explored. Considering the clinical and economic burden of readmissions, evaluating whether SII can serve as an accessible and cost‐effective predictor may offer meaningful value for risk stratification. Therefore, this study aims to investigate the association between SII and hospital readmission rates in patients with AMI, addressing a gap in the current literature.

## 2. Materials and Methods

### 2.1. Study Population and Design

This single‐center, retrospective cohort study examined 1147 patients diagnosed with AMI according to the International Classification of Diseases, 10th Edition (ICD‐10). The patients were admitted to Dr. Heshmat Hospital, affiliated with Guilan University of Medical Sciences (GUMS) in Rasht, Iran, between 2019 and 2021. An MI, according to the Fourth Universal Definition, is characterized as an acute myocardial injury accompanied by clear clinical evidence of acute myocardial ischemia, demonstrated by ischemic symptoms, new ECG abnormalities such as pathological Q‐waves, or imaging findings showing new loss of viable myocardium or new regional wall‐motion abnormalities consistent with an ischemic cause [16].

Patients who had routine blood tests available and presented on the first day of hospitalization were included in the study. Patients whose medical records and laboratory results were incomplete in the hospital’s system were excluded from the study. The study met the 2013 guidelines of the Declaration of Helsinki, and the protocol received approval from the Institutional Review Board of the Cardiovascular Diseases Research Center at GUMS and the GUMS Ethics Committee (ethics code: IR.GUMS.REC.1402.380).

### 2.2. Clinical and Laboratory Data

Demographic and clinical data including age, gender, smoking status, comorbidities (including hypertension [HTN], hyperlipidemia [HLP], and diabetes mellitus [DM]), family history of CVDs, medical history (including CVD, percutaneous coronary intervention [PCI], and coronary artery bypass grafting [CABG]), performing coronary intervention during hospitalization (PCI or coronary angiography [CAG]), and their results (the number of diseased vessels) were gathered retrospectively from patients’ medical records. Laboratory findings including the level of platelet, neutrophil, lymphocyte, blood urea nitrogen (BUN), creatinine (Cr), WBC, hemoglobin, cholesterol, low‐density lipoprotein (LDL), high‐density lipoprotein (HDL), and triglyceride were recorded from the medical system.

### 2.3. Outcomes

For all patients, data on primary outcomes including major adverse cardiovascular events (MACEs) defined as a composite of recurrent myocardial infarction (re‐MI), stroke, and cardiovascular death, as well as secondary outcomes including embolic events, stable and unstable angina, congestive HF (which was defined as hospitalization due to clinical signs and symptoms of volume overload [e.g., dyspnea, pulmonary rales, and peripheral edema] confirmed by physician diagnosis and supported by echocardiographic or laboratory findings when available), or other relevant conditions were collected up to 4 years after initial hospitalization.

Additionally, the readmission date was documented. In cases where patients had not returned to our center or their readmission status was unclear, they were contacted via phone. If they had been admitted to other medical facilities, the admission date and its reason were recorded accordingly. In this study, readmission was defined as all‐cause hospitalization, regardless of cardiovascular or noncardiovascular etiology.

### 2.4. SII Calculation

SII was calculated as total peripheral platelets count (P) × neutrophil‐to‐lymphocyte ratio (N/L) (SII = P × N/L ratio) [17]. Based on a recent study [18], optimal cutoff point for SII was determined as 694.3 × 10^9^/L with high SII considered as ≥ 694.3 and low SII considered as < 694.3.

### 2.5. Bias Considerations

To minimize selection bias, only patients with complete clinical and laboratory records were included. Clinical and laboratory data were extracted from standardized electronic medical records, reducing the risk of information bias. Diagnostic criteria for AMI were consistently applied based on ICD‐10 codes and validated biomarkers. Potential confounding variables identified in preliminary analyses were adjusted for using multivariate Cox regression models. Outcome data (readmission) were verified through medical records and follow‐up calls, minimizing observer bias through standardized documentation.

### 2.6. Sample Size Calculation

This study included all patients admitted to our hospital with AMI between 2019 and 2021 whose medical records were complete (*n* = 1147).

### 2.7. Statistical Analysis

In this study, the normality of quantitative variables had been assessed using the Kolmogorov–Smirnov test. If the data were found to be normally distributed, the variables had been reported using the mean and standard deviation; otherwise, the median and interquartile range had been used. In two groups: If the data were normally distributed, an independent *T*‐test had been performed; otherwise, the Mann–Whitney test had been applied. In three or more groups: If normality was confirmed, an ANOVA test had been conducted; otherwise, the Kruskal–Wallis test had been used. Categorical variables: These variables had been reported using frequency and percentage, and comparisons had been made using either the chi‐square test or Fisher’s exact test. To illustrate patient survival rates based on primary and secondary outcomes across different groups, the Kaplan–Meier curve had been employed. A univariate Cox proportional hazards model had been used to examine the relationship between SII and primary and secondary outcomes. If confounding variables had been identified in preliminary analyses, an adjusted Cox model had been applied. A significance level of 0.05 had been considered for all tests. Data analysis had been performed using IBM SPSS (Version 22) and R software when necessary.

## 3. Results

In this retrospective cohort study, 1147 AMI patients were investigated. Optimal cutoff point for SII was determined as 694.3 × 10^9^/L with high SII considered as ≥ 694.3 and low SII considered as < 694.3. The mean age of the patients was 60.3 (12.18) years which was higher in high SII patients (*p* < 0.05). 27.5% of patients were female. The incidence of HTN, DM, and HLP was 43.9%, 29.8%, and 29.8%, respectively. 62.5% of patients underwent PCI during hospitalization. Patients with higher SII had higher platelet (224.5 × 10^3^), neutrophil (80.5 × 10^9^), WBC (11500), and BUN [17]. Patients with high SII had higher prevalence of PECA (9.7%), 2VD (26.6%), and three‐vessel disease (3VD) (32%) compared with low SII; however, these differences were not significant (*p* > 0.05) (Table 1).

**TABLE 1:** Distribution of demographic and clinical characteristics of studied population according to SII cutoff.

Variable		All	SII < 694.3	SII ≥ 694.3	*p* value
		*N* = 1417	*N* = 955	*N* = 462	
Age	Mean (SD)	60.3 (12.2)	59.7 (12.1)	61.4 (12.4)	0.015^#^
	Median (IQR)	60.0 (52.0–68.0)	59.0 (52.0–67.0)	61.0 (54.0–69.0)	

Gender, *n* (%) (female)		390 (27.5)	239 (25.0)	151 (32.7)	0.002^∗^

Smoking, *n* (%)		488 (34.4)	334 (35.6)	154 (33.4)	0.417^∗^

Comorbidities *n* (%)	HTN	622 (43.9)	419 (44.7)	203 (44.0)	0.822^∗^
	DM	422 (29.0)	294 (31.3)	128 (27.8)	0.171^∗^
	HLP	422 (29.8)	287 (30.6)	135 (29.3)	0.615^∗^
	CVDs	195 (14.0)	135 (14.4)	60 (13.1)	0.490^∗^

Family history of CVD, *n* (%)		382 (27.4)	259 (27.7)	123 (26.8)	0.730^∗^

Intervention history *n* (%)	PCI	110 (7.9)	76 (8.1)	34 (7.4)	0.650^∗^
	CABG	104 (7.5)	80 (8.5)	24 (5.2)	0.027^∗^

PCI in hospitalization, *n* (%)		891 (62.9)	605 (63.4)	286 (61.9)	0.590^∗^

CAG in hospitalization, *n* (%)		1235 (87.2)	832 (87.1)	403 (87.2)	0.950^∗^

Intervention results, *n* (%)	PECA	134 (9.5)	89 (9.3)	45 (9.7)	0.540^∗^
	Mild CVD	53 (3.7)	41 (4.3)	12 (2.6)	
	SVD	352 (24.8)	243 (25.4)	109 (23.6)	
	2VD	349 (24.6)	226 (23.7)	123 (26.6)	
	3VD	450 (31.8)	302 (31.6)	148 (32.0)	

Laboratory parameters	Platelet (10^9^/L)				
	Mean (SD)	214.8 (64.7)	201.4 (55.6)	239.5 (72.7)	< 0.001^∗∗^
	Median (IQR)	204.0 (170.0–247.0)	192.0 (161.0–232.0)	224.5 (188.7–278.2)	
	Neutrophil (10^9^)				
	Mean (SD)	70.3 (13.5)	62.5 (12.2)	79.7 (7.8)	< 0.001^∗∗^
	Median (IQR)	72.0 (63.0–80.0)	65.0 (57.0–70.0)	80.5 (75.0–85.0)	
	Lymphocyte (10^9^/L)				
	Mean (SD)	23.7 (10.9)	30.7 (9.3)	15.4 (5.7)	< 0.001^∗∗^
	Median (IQR)	22.0 (15.0–31.0)	30.0 (25.0–37.0)	15.0 (11.0–19.0)	
	BUN				
	Mean (SD)	18.7 (8.9)	18.3 (8.1)	19.5 (10.2)	0.090^∗∗^
	Median (IQR)	16.0 (14.0–20.0)	16.0 (14.0–20.0)	17.0 (14.0–21.0)	
	Cr				
	Mean (SD)	1.2 (0.8)	1.2 (0.6)	1.2 (1.2)	0.330^∗∗^
	Median (IQR)	1.1 (0.9–1.2)	1.1 (0.9–1.2)	1.0 (0.9–1.3)	
	WBC				
	Mean (SD)	10807.9 (3835.7)	10078.5 (3481.4)	12159.1 (4090.8)	< 0.001^∗∗^
	Median (IQR)	10100.0 (8100.0–12700.0)	9500.0 (7700.0–11700.0)	11500.0 (9350.0–14100.0)	
	Hemoglobin				
	Mean (SD)	13.4 (2.1)	13.4 (2.0)	13.3 (2.2)	0.230^∗∗^
	Median (IQR)	13.5 (12.1–14.7)	13.6 (12.3–14.7)	13.3 (11.9–14.7)	
	Cholesterol				
	Mean (SD)	165.2 (51.4)	165.4 (48.9)	164.8 (56.0)	0.450^∗∗^
	Median (IQR)	158.0 (133.0–190.5)	160.0 (134.0–192.0)	157.0 (131.0–189.0)	
	LDL				
	Mean (SD)	101.7 (39.6)	102.1 (37.9)	100.5 (43.9)	0.510^∗∗^
	Median (IQR)	97.8 (76.2–123.1)	99.2 (76.6–124.2)	95.2 (75.2–123.1)	
	HDL				
	Mean (SD)	39.2 (17.1)	39.3 (18.2)	38.8 (13.5)	0.890^∗∗^
	Median (IQR)	38.0 (31.0–44.0)	38.0 (31.0–44.0)	38.0 (30.0–45.0)	
	TG				
	Mean (SD)	146.6 (96.5)	150.9 (103.6)	138.3 (81.0)	0.024^∗∗^
	Median (IQR)	122.5 (88.0–173.0)	126.0 (91.0–178.0)	116.0 (85.0–164.0)	

*Note:* *p* value < 0.05 was considered as significance level.

Abbreviations: BUN, blood urea nitrogen; CAG, coronary angiography; CABG, coronary artery bypass grafting; Cr, creatinine; CVDs, cardiovascular diseases; DM, diabetes mellitus; HLP, hyperlipidemia; HDL, high‐density lipoprotein; HTN, hypertension; LDL, low‐density lipoprotein; PCI, percutaneous coronary intervention; TG, triglyceride; WBC, white blood cell count.

^∗^Chi‐square.

^∗∗^Mann–Whitney.

^#^Independent *T*‐test.

Over a 4‐year period, 454 patients were readmitted to the hospital for various reasons. Among them, those with high SII had a higher rate of hospital readmission as an endpoint (34.4%), although it was not statistically significant. The study’s primary outcomes included death, stroke, and re‐MI, with 97 patients being rehospitalized due to these factors. Notably, the incidence of re‐MI was the most frequent among primary outcomes (5.2%). Statistically significant differences were observed in the occurrence of primary outcomes based on SII cutoffs, with patients having higher SII experiencing a greater incidence of primary outcomes (*p* < 0.05). Additionally, 404 patients were readmitted over four years due to secondary outcomes, with unstable and stable angina being more prevalent in the high SII group, though these differences were not statistically significant (*p* > 0.05) (see Table 2).

**TABLE 2:** Clinical outcomes in participants based on the SII score.

Outcomes	Category	All *N* = 1417	SII < 694.3	SII ≥ 694.3	*p* value
			*N* = 955	*N* = 462	
Readmission		454 (32.0)	295 (30.9)	159 (34.4)	0.182^∗^

Primary outcome	Death	31 (2.7)	13 (1.7)	18 (5.2)	0.006^∗∗^
	Stroke	7 (0.6)	5 (0.6)	2 (0.6)	
	Re‐MI	59 (5.2)	37 (4.7)	22 (6.3)	

Secondary outcome	Embolic events	5 (0.4)	4 (0.5)	1 (0.3)	0.611^∗^
	Heart failure	39 (3.5)	27 (3.5)	12 (3.5)	
	Stable angina	39 (3.5)	23 (3)	16 (4.6)	
	Unstable angina	54 (4.8)	34 (4.4)	20 (5.8)	
	Others	303 (27.0)	209 (26.9)	94 (27.2)	

*Note:* *p* value < 0.05 was considered as significance level. Data were presented as number (percent).

Abbreviation: MI, myocardial infraction.

^∗^Chi‐square.

^∗∗^Fisher’s exact test.

**FIGURE 1:**
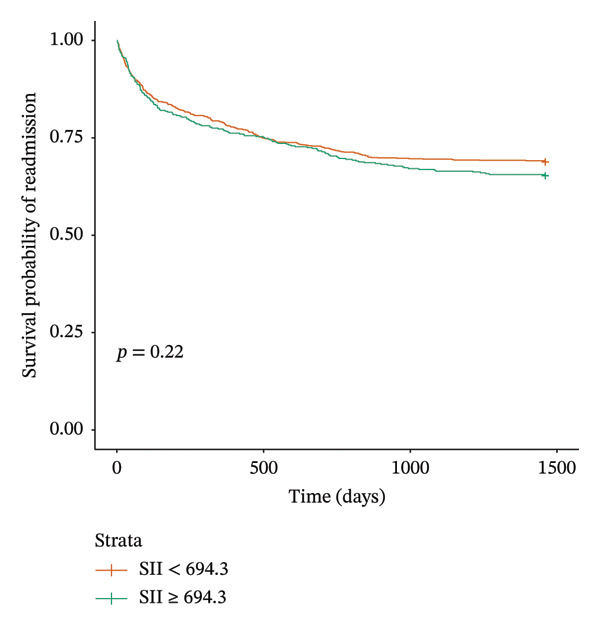
Kaplan–Meier survival curve analysis of readmission.

The log‐rank test and Kaplan–Meier survival analysis suggest that there is no association between SII and readmission within a maximum of 4 years following the initial hospitalization (Figure 1).

The Cox proportional hazards model was utilized to investigate the impact of high SII on the readmission within 4 years following the initial hospitalization, adjusting variables that exhibited differences in the preliminary analysis presented in Table 1 between the two SII groups. In Model 1 and Model 2, individuals who underwent prior CABG have a 40% higher hazard of readmission compared to others (see Table 3).

**TABLE 3:** The association of SII and readmission adjusting age, gender, prior CABG, WBC, and triglyceride.

	Model 1 HR (95% CI)	Model 2 HR (95% CI)	Model 3 HR (95% CI)
SII ≥ 694.3	1.12 (0.92–1.36)	1.13 (0.93–1.37)	1.14 (0.92–1.42)
Age	1.00 (0.99–1.01)	1.01 (0.99–1.01)	1.01 (0.99–1.01)
Gender (male)	1.02 (0.83–1.26)	1.02 (0.82–1.25)	1.04 (0.83–1.31)
Prior CABG	—	1.43 (1.04–1.95)	1.44 (1.02–2.03)
WBC	—	—	1.00 (1.00–1.00)
Triglyceride	—	—	0.99 (0.99–1.001)

Abbreviations: CABG, coronary artery bypass graft surgery; WBC, white blood cell count.

## 4. Discussion

To the best of our knowledge, this is the first study to specifically evaluate the association between SII and long‐term hospital readmission in patients with AMI. Although SII has been widely investigated as a prognostic marker for mortality, MACE, and adverse cardiovascular outcomes, its potential role in predicting readmission, an outcome with major clinical and economic implications, has remained largely unexplored. By focusing on 4‐year readmission trends, our study addresses this important gap in the literature. Our findings showed that patients in the high SII group were generally older and more often female, and they exhibited higher platelet, neutrophil, WBC, and BUN levels. Crude readmission rates were also higher in the high SII group, primarily due to re‐MI. SII did not demonstrate a statistically significant association with readmission. Notably, SII was not associated with readmission in the crude analysis, and this lack of significance persisted in both Kaplan–Meier survival curves and the multivariable Cox regression model. These results indicate that, despite the numerically higher readmission frequency observed in the high‐SII group, SII did not independently predict long‐term readmission in our cohort.

AMI initiates an inflammatory response crucial for cardiac repair. Beyond localized myocardial inflammation, patients with AMI also exhibit heightened systemic inflammatory activity [3]. Atherosclerosis is an inflammatory disease where leucocytes, lymphocytes, and platelets’ reactivity might have a central role [19, 20]. Atherosclerosis, an inflammatory condition, involves key interactions between leucocytes, lymphocytes, and platelets, contributing to disease progression. While certain inflammatory markers such as neutrophil‐to‐lymphocyte ratio (NLR), platelet‐to‐lymphocyte ratio (PLR), and monocyte‐to‐lymphocyte ratio (MLR) have been investigated for prognosis estimation in STEMI patients, their predictive power remains limited [21, 22]. A recent study reported that an elevated Inflammatory Burden Index derived from CRP, neutrophil count, and lymphocyte count was strongly associated with 30‐day readmission after elective PCI, independent of conventional clinical risk factors. These findings indicate that IBI may be a useful marker for identifying patients who are at higher risk of early rehospitalization [23]. Evidence suggests that SII is an independent predictor of mortality (cardiovascular and all‐cause) in the ACS population. Additionally, it effectively stratifies the risk of hospital readmission related to either MACE [18, 25] or other adverse outcomes [24]. Notably, SII has demonstrated strong associations with both cardiovascular and all‐cause mortality in diabetic patients [26]. A recent study suggested that incorporating SII into a regression model with HDL and LDL may serve as a potential diagnostic indicator for ACS in patients with diabetes or prediabetes presenting with chest pain [27]. Peripheral lymphopenia during MI has been identified as a marker of severe disease progression, while increased platelet counts may correlate with adverse outcomes due to heightened inflammatory mediator release. Excessive platelet activation exacerbates inflammatory responses, fostering a prothrombotic state [28]. Additionally, neutrophil extracellular traps contribute to multiple prothrombotic effects, including platelet adhesion, activation, and aggregation, further complicating disease pathology [29].

Multiple studies have linked SII to adverse outcomes in CVDs patients, including chronic HF, atrial fibrillation, and peripartum cardiomyopathy [30–32]. Additionally, SII has been independently associated with contrast‐induced nephropathy following PCI in non‐STEMI patients, coronary collateral circulation formation in stable CAD, and mortality in chronic HF patients [30, 33, 34]. Consistent with our findings, research has demonstrated that elevated SII correlates with MACCE incidence and long‐term mortality in patients with 3VD after revascularization [35]. Moreover, a linear positive relationship has been observed between SII levels and hospital readmissions in individuals experiencing acute exacerbations of bronchiectasis [36].

Our study further revealed that patients with higher SII were generally older compared to those in the low SII group. Age plays a crucial role in immune and inflammatory responses, with younger individuals maintaining a more balanced inflammatory and anti‐inflammatory state, whereas older patients are more susceptible to dysregulation [37]. Additionally, in our study, gender differences were evident, with female patients exhibiting higher SII levels. Research suggests that inflammatory responses vary between males and females [38], largely influenced by reproductive hormones, testosterone in males and estrogen in females [38, 39]. Furthermore, studies indicate that gender can impact immune cell function, particularly affecting neutrophils and lymphocytes [40]. These findings, along with our own results, emphasize the clinical relevance of SII as a predictive marker in cardiology, particularly for high‐risk cardiovascular patients.

Taken together, these findings highlight the novelty of our work: While previous studies have established SII as a predictor of mortality, MACE, and other adverse cardiovascular outcomes, recent evidence from 2023 to 2025 has further emphasized the growing importance of inflammatory biomarkers in AMI risk stratification. However, despite these contemporary advances, no recent study has specifically examined the role of SII in predicting long‐term hospital readmission, an outcome increasingly recognized as a key quality‐of‐care indicator with major clinical and economic implications. Although our dataset originates from 2021, the inflammatory pathways reflected by SII remain biologically stable and clinically relevant. By evaluating 4‐year readmission trends, our study fills this persistent gap in the literature and extends the clinical relevance of SII beyond mortality and MACE to the domain of recurrent hospitalization, offering a simple and accessible tool for identifying high‐risk AMI.

## 5. Strengths and Limitations

Our study had some strengths and limitations, which should be noted. The inclusion of 1147 patients provides a robust dataset for analysis, enhancing the reliability of findings. Moreover, using hospital records ensures the study reflects real‐world patient outcomes, making the results clinically relevant. The other strength of this study is the efforts which were made to contact patients who were not readmitted to the same hospital, improving data completeness.

The retrospective design of this study can be considered as a limitation which relies on existing medical records, making it prone to missing or incomplete data. Patients were recruited from one hospital (Dr. Heshmat Hospital, as specialized heart center in the north of Iran), limiting generalizability to broader populations. While attempts were made to contact patients admitted elsewhere, accurate tracking of their medical events may not be comprehensive. Furthermore, the SII was not evaluated alongside other inflammatory markers such as C‐reactive protein (CRP), NL, PLR, and MLR. Given these limitations, future prospective studies involving larger and more diverse populations, as well as a comparative analysis of multiple inflammatory indicators, are essential to substantiate our findings.

## 6. Conclusion

In conclusion, although patients with high SII experienced higher crude rates of readmission particularly due to re‐MI, SII did not demonstrate a statistically significant association with readmission. These findings indicate that SII reflects inflammatory burden but does not independently predict long‐term readmission after AMI when major clinical confounders are considered. Larger prospective studies are needed to further clarify the potential role of SII in post‐AMI risk stratification.

## Author Contributions

Yasaman Borghei and Arsalan Salari: conception and design. Nasibe Goli and Fatemeh Baharvand: acquisition of data and material preparation. Bahare Gholami‐Chaboki: analysis of data. Yasaman Borghei, Fatemeh Baharvand, and Arsalan Salari: writing, review, and revision of the manuscript.

## Funding

No fund was received for this study.

## Disclosure

All authors have read and approved the final manuscript.

## Conflicts of Interest

The authors declare no conflicts of interest.

## Data Availability

The data supporting the findings of this study are available from the corresponding author upon reasonable request. They are not publicly accessible due to privacy and ethical restrictions.
